# A Rare Case of Aggressive Atypical Cervical Cancer With Multi-Organ Involvement

**DOI:** 10.7759/cureus.32968

**Published:** 2022-12-26

**Authors:** Carina Hernandez, Alanna Glidden, Michael Sandhu, Kavita Agrawal, Martha Caicedo Murillo, Claudia Heritage, Merima Ramovic, Komal Akhtar

**Affiliations:** 1 Internal Medicine, Upstate University Hospital, Syracuse, USA; 2 Hematology and Oncology, Upstate University Hospital, Syracuse, USA; 3 Pathology, Upstate University Hospital, Syracuse, USA

**Keywords:** hpv, multi-organ, brain metastasis, squamous cell carcinoma, cervical cancer

## Abstract

Squamous cell carcinoma (SCC) of cervical origin with metastasis to the brain is rare. Our patient was a 30-year-old Caucasian female with squamous cell carcinoma, initially with unknown primary, with metastases to the brain, kidney, cervix, lung, adrenal glands, vulva, pelvic wall, and scalp. She initially presented to her outpatient gynecologist for a vulvar mass. A biopsy of the vulvar mass was consistent with SCC. The patient continued to have fatigue along with thoracic rib pain. An initial work-up was performed, including imaging which showed diffuse metastatic disease involving the lungs, kidneys and adrenal glands, as well as a pathological compression fracture of the seventh thoracic vertebra with cord compression. Brain magnetic resonance imaging (MRI) showed multiple metastatic lesions and she underwent craniotomy for brain lesion resection. Given the aggressive nature of the patient’s disease and her symptomatic burden, she was started on chemotherapy in the hospital with Carboplatin, Paclitaxel, and Pembrolizumab.

## Introduction

The fourth most common malignancy worldwide in women is cervical cancer [1]. The predominant type of cervical cancer is squamous cell carcinoma, other subtypes being adenocarcinoma and adenosquamous carcinoma. The greatest risk factor for cervical cancer is infection with human papillomavirus (HPV) [2]. Other risk factors include smoking, oral contraceptive use, and diet [3]. High parity has been positively associated with cervical cancer as well [4]. It is most commonly found in women aged 35 to 54 years of age and can usually be detected in the early stages with a Pap test. The updated cervical cancer screening guidelines from the American Cancer Society recommend starting screening at age 25 and HPV test every five years [5].

Metastatic squamous cell carcinoma arising from the cervix and metastasizing to the brain is very rare, reportedly as low as 0.4% to 2.3% [6, 7] and 2-8% in patients that already have spread to extracranial sites [8]. The risk of metastatic disease to the brain is increased when tumors are poorly differentiated. Brain lesions are often supratentorial, likely due to vascularity and spatial characteristics of the area. The prognosis of cancer that has metastasized to the brain is very poor, with the mean survival of two to eight months [9]. A multi-modal treatment approach, consisting of chemotherapy, surgery, and radiation provides possible options for the prolongation of life [6].

We present a unique case of a 30-year-old female with multiple metastases, including intracranial metastasis and malignant spinal cord compression from cervical cancer, which is rare.

## Case presentation

A 30-year-old female with no significant past medical history, initially presented to her outpatient gynecologist for a vulvar mass. A biopsy showed non-keratinizing squamous cell carcinoma with areas of extensive necrosis. She then presented to an outside hospital a week later with weakness, fatigue, difficulty with ambulation, back pain, and thoracic rib pain. A computed tomography (CT) showed diffuse metastatic disease involving the lungs, kidneys, and adrenal glands. She was sent home from the emergency room and advised to follow up with oncology as soon as possible. The next day, she presented to our institution with worsening back pain.

**Figure 1:**
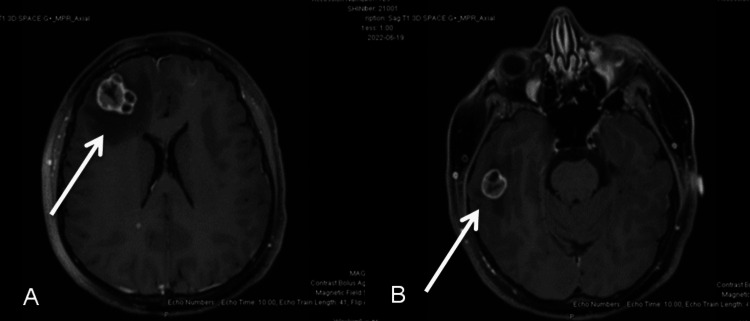
Pre-operative MRI axial image weighted in T1 showing lesions. There were multiple variable-sized enhancing lesions in the supratentorial (A and B) and infratentorial brain parenchyma with surrounding vasogenic edema likely representing metastasis. (A) There was a 2.7 x 2.6 cm lesion in the right anterior frontal lobe with moderate surrounding vasogenic edema with mass effect upon the underlying cerebellar hemisphere and effacement of the right lateral ventricle and also causing mild subfalcine herniation. (B) A 1.9 cm lesion was seen in the right inferior temporal lobe.

**Figure 2:**
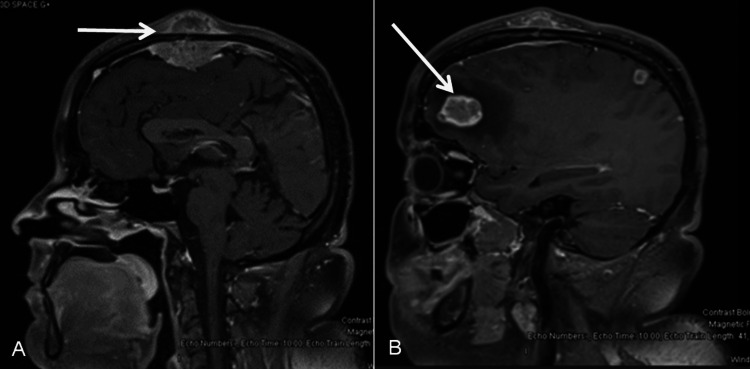
Pre-operative MRI of the head sagittal T1 image showing metastatic lesions. There were enhancing dural-based extra-axial lesions in the parafalcine location with involvement of the calvarium (A) and enhancing adjacent soft tissue component and causing encasement of the superior sagittal sinus representing metastasis. (B) There was a 2.7 x 2.6 cm lesion in the right anterior frontal lobe with moderate surrounding vasogenic edema with mass effect.

A metastatic workup was completed. MRI of the brain showed a large right frontal lobe lesion measuring 2.7 x 2.6 cm (Figure 1) with vasogenic edema, right parietal lobe, right temporal lobe, and left posterior cerebellar hemisphere lesions, as well as a dural-based extra-axial lesion in the parafalcine location with an adjacent soft tissue lesion measuring 2.8 x 1.1 cm (Figure 2). CT abdomen and pelvis showed multiple lung base pulmonary masses, hypodensities in the liver, left renal masses, enlarged uterine cervix and other metastases including adrenal glands, and pelvic side wall. A repeat CT thorax was obtained to better examine the pulmonary lesions. It showed large masses at the left hilum, posterior right upper lobe, posterior inferior right lower lobe with significant extension into the mediastinum, contiguous with the lateral wall of the aorta, pulmonary artery, and compressing the left upper lobe and lingular bronchus. The CT also showed multiple soft tissue nodules of the chest and breast. The bone scan showed increased activity within the left tibial shaft, right proximal femoral shaft, and skull, with lytic changes seen in the right scapula. MRI spine showed a severe compression fracture of T7 with compression of the spinal cord.

**Figure 3:**
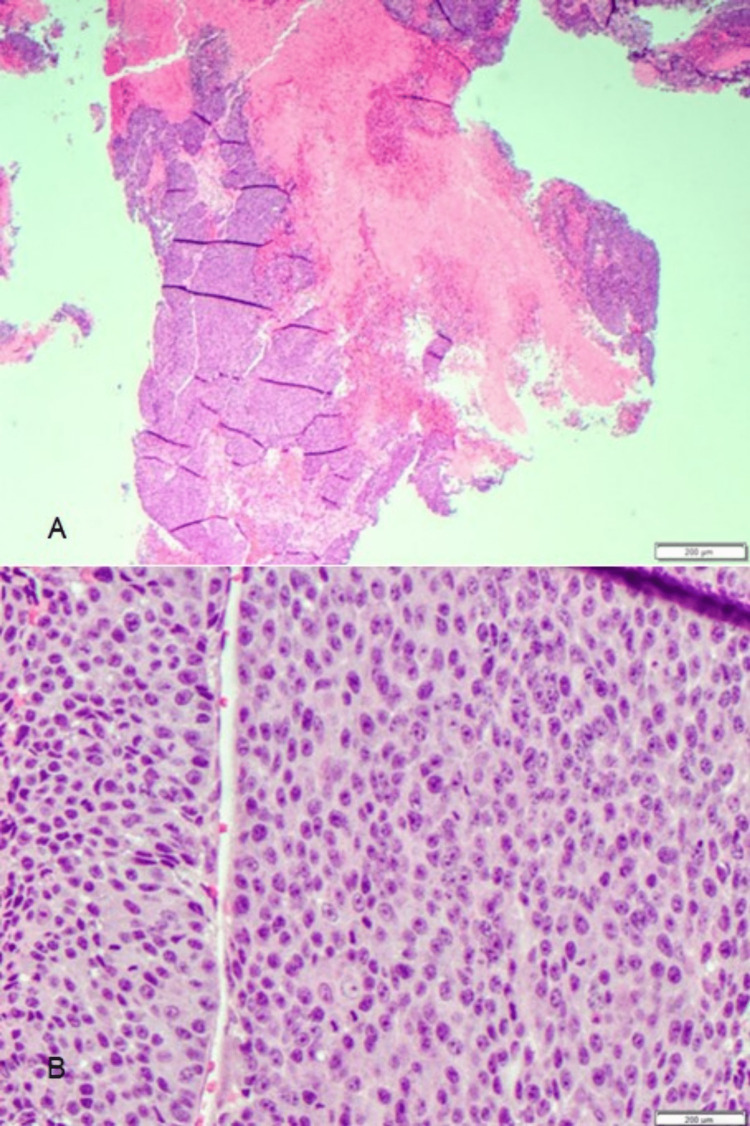
Squamous cells from spinal tumor biopsy. (A) Low power view showing multiple fragments of poorly differentiated squamous cells and prominent necrosis. (B) High power view showing features of the squamous cells which are pleomorphic, has prominent nucleoli, eosinophilic cytoplasm and frequent mitosis; similar features seen on the vulvar lesion.

**Figure 4:**
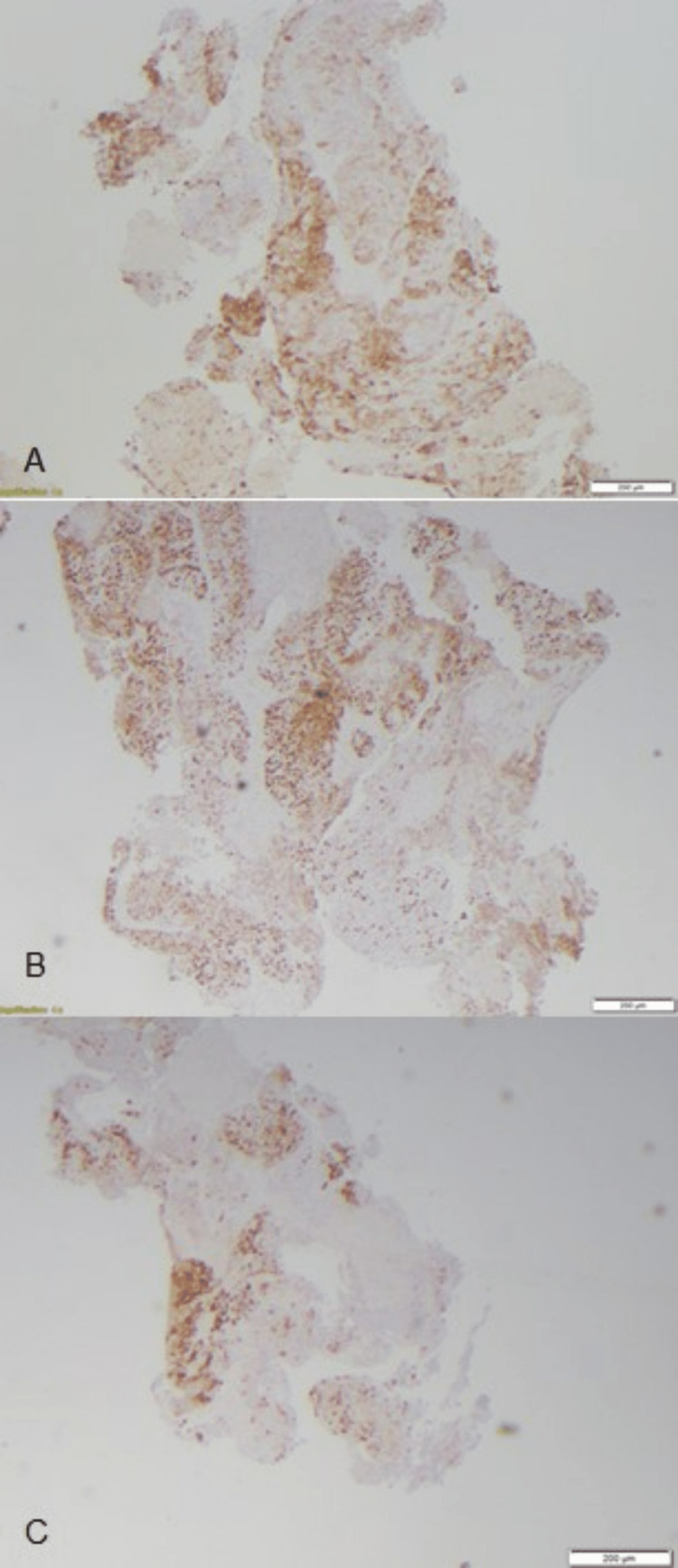
Immunohistochemical analysis of spinal tumors specimen. Immunohistochemistry of the resected tissue specimen was positive for cytokeratin 5/6 or CK5/6 (A), caudal-type homeobox protein 2 or CDX2 (B), and variable positivity for p16 (C).

She underwent a laminectomy of T7 with tumor resection and T4-T9 fusion by neurosurgery for cord compression. Histopathological examination of the resected spinal tumor revealed poorly differentiated squamous cell carcinoma with necrosis. Immunohistochemistry showed CK5/6+, CDX2+, and variable p16 suggestive of genital origin (Figure 3 and Figure 4).

**Figure 5:**
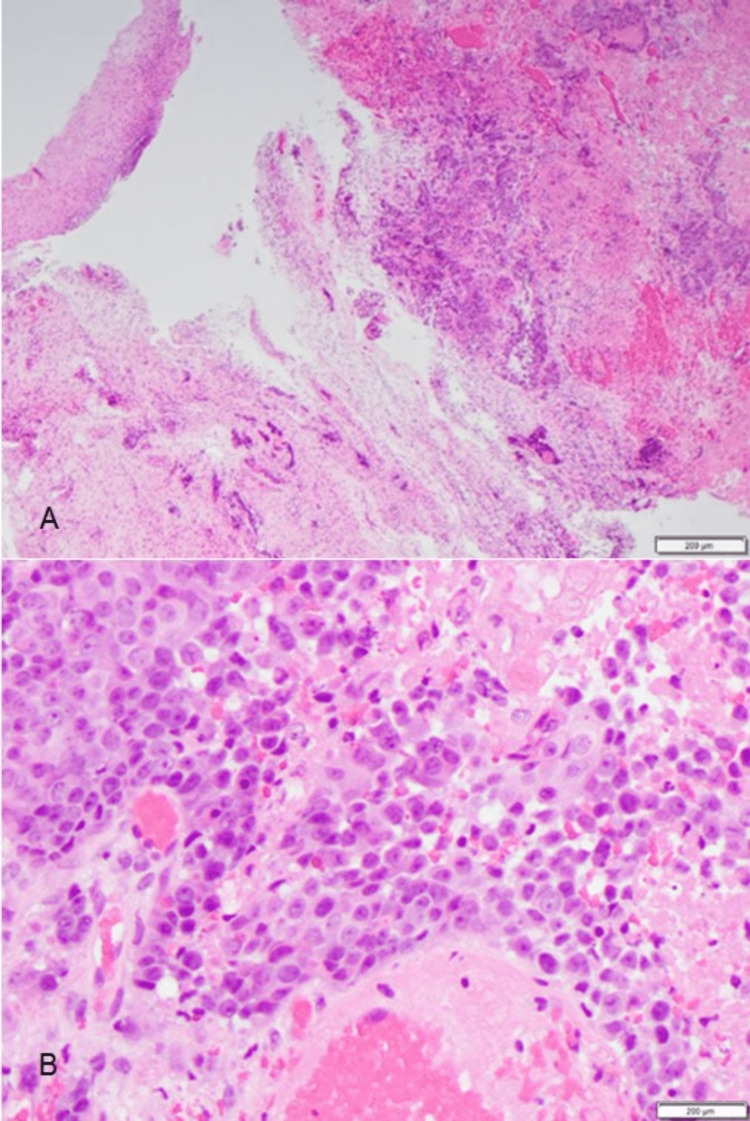
Surgical pathology for anterior cervical biopsy. The image shows that the tumor involves stroma and does not connect to overlying histologically unremarkable squamous mucosa. The tumor may represent metastasis or direct extension of the tumor from the adjacent site. Tumor necrosis is seen. (A) Low power view: Right tumor involving the stroma, Left, unremarkable ectocervix. (B) High power view showing cells reminiscent of squamous cells.

**Figure 6:**
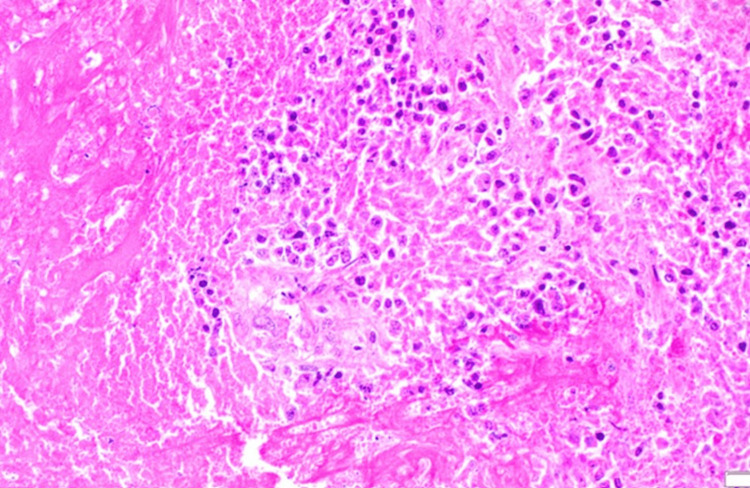
Anterior cervix and endocervical canal. Cytology cell block shows predominantly necrotic debris and cells with prominent nucleoli and high nuclear-to-cytoplasmic ratio.

She underwent a left radical vulvectomy by gynecology. A 4 cm lesion was removed from the left labia majora (Figure 5 and Figure 6). Histopathologic findings from the vulvar lesion showed squamous and neuroendocrine differentiation, with positive CK7, CDX2 and SATB-2. Ki-67 proliferation index was greater than 90%. Immunostains (p16, p63, CK5/6 and CK7) were used in the evaluation confirming squamous cell carcinoma (SCC). The tumor demonstrated P16 staining in 50 to 60% of the tumor. Upon HPV in situ hybridization for high-risk HPV 16/18 was negative. Additionally, the tumor was sent to Memorial Sloan Kettering Cancer Center (MSKCC) for NUT (nuclear protein in testis) carcinoma immunostaining which was also negative. The anterior cervical biopsy and recent Pap smear showed similar findings of poorly differentiated SCC.

The patient had complaints of fatigue, weakness, and trouble ambulating. On imaging, she had multiple brain metastases, including a large right frontal tumor that was not amenable to radiation due to size. The right anterior frontal lobe had moderate surrounding vasogenic edema with mass effect upon the underlying cerebellar hemisphere and effacement of the right lateral ventricle causing mild subfalcine herniation. Additionally, she had a midline parietal lesion with both an extracranial and intracranial component and the extracranial component was causing emotional distress due to its prominence.

**Figure 7:**
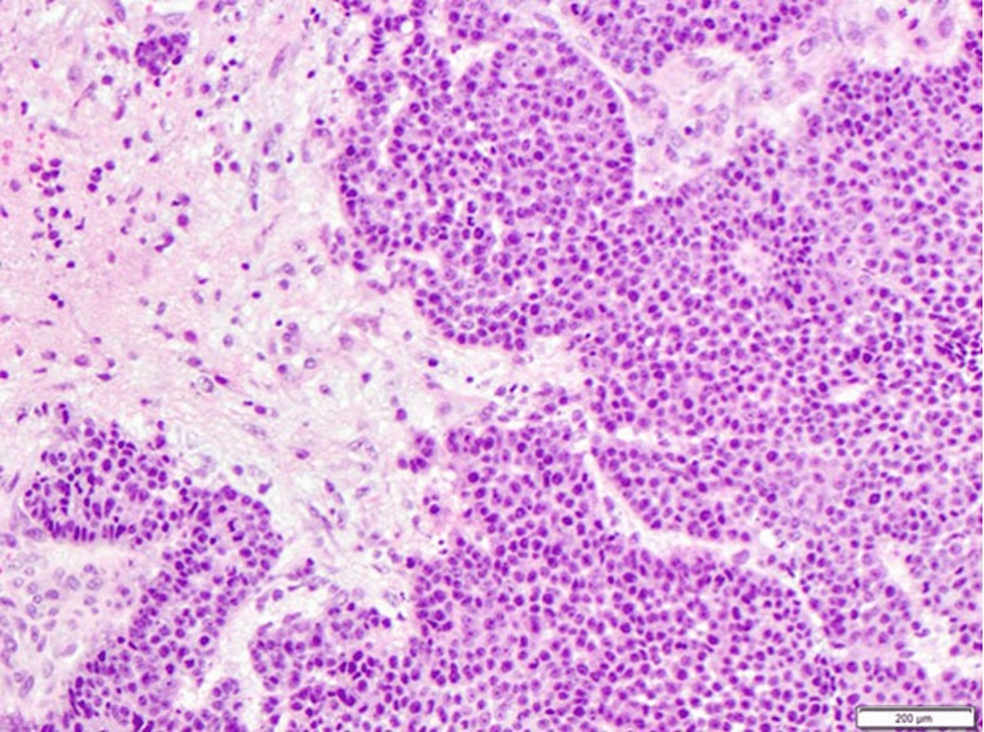
Surgical pathology of intracranial lesion. The left area shows what resembles a normal brain. The right shows prominent poorly differentiated squamous cells.

**Figure 8:**
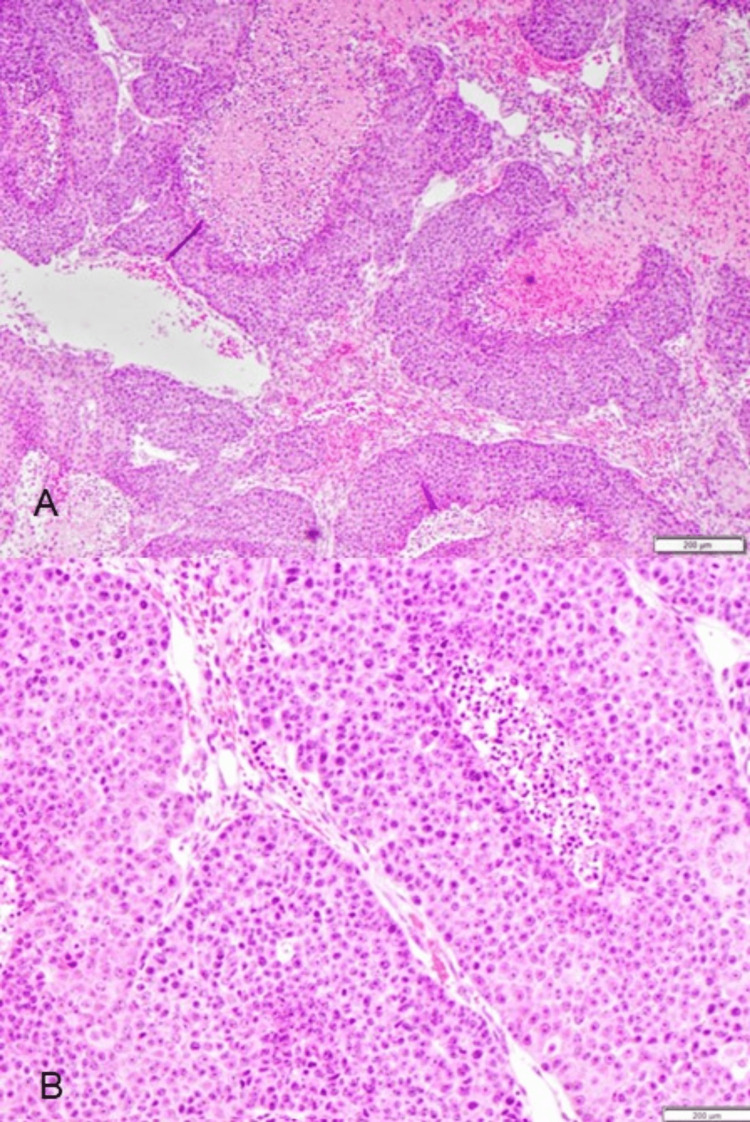
Surgical pathology of extracranial lesion. (A) This low-power image shows poorly differentiated squamous carcinoma with prominent “comedo-like” necrosis. (B) High power view showing abundant necrosis, pleomorphism, prominent nucleoli, and eosinophil cytoplasm.

The patient had craniotomy and tumor resection of the right frontal intra-axial tumor (Figure 7), as well as resection of the midline frontal extracranial tumor (Figure 8). Histopathological examination of the intracranial and extracranial lesions indicated metastatic, poorly differentiated carcinoma that was morphologically similar to the patient's spinal tumor and vulvar mass.

**Figure 9:**
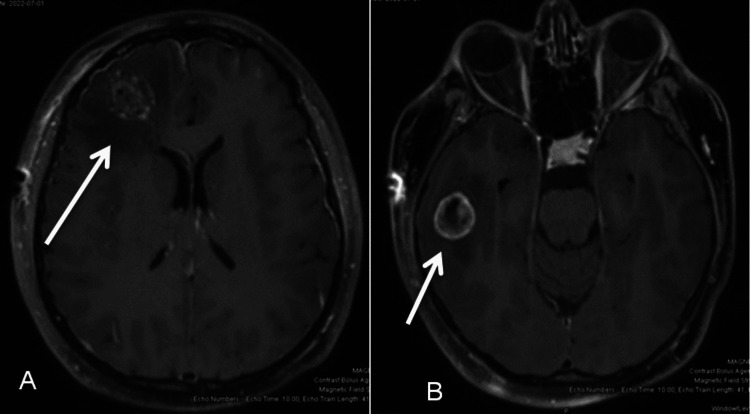
Post-operative MRI of the patient’s brain axial image weighted in T1 showing metastatic lesions. There was a moderate amount of right frontal vasogenic edema and an adjacent enhancing (A) lesion at the superior medial aspect of the surgical bed that increased in size compared to the prior study consistent with neoplasm. (B) There are multiple bilateral enhancing cerebral and cerebellar lesions.

MRI brain performed post-craniotomy showed enhancing lesions at the superior medial aspect of the surgical bed that increased in size compared to the prior study (Figure 9). There were multiple bilateral enhancing cerebral and cerebellar lesions. There were also multiple calvarial, scalp, and subdural metastases causing mass effect.

The patient’s blood work demonstrated that she had elevated levels of Cancer Antigen 27-29 (CA27-29) and Cancer Antigen 125 (CA-125). However, Cancer Antigen 15-3 (CA15-3), Cancer Antigen 19-9 (CA19-9), and Carcinoembryonic Antigen (CEA) were all within normal limits.

With the immunohistochemistry suggesting metastatic squamous cell carcinoma, likely of cervical origin, it was decided to give her at least one dose of Carboplatin, Paclitaxel, and Pembrolizumab inpatient following the cervical cancer pathway as well as the squamous histology, while she was waiting to begin post-op RT to brain and to T spine. At the time PD-L1 staining was not done.

However, due to the severe tumor burden, her disease progressed. She became encephalopathic due to worsening neoplastic disease. A repeat MRI brain showed marked interval progression of metastatic disease with numerous new lesions and interval increase of previous lesions. Whole brain radiation therapy was attempted however, the patient was unable to tolerate. The patient and her family decided to focus on palliative measures and she was discharged home, where she passed away shortly thereafter.

## Discussion

The fourth most common malignancy after breast cancer in women is cervical cancer. This cancer is usually seen in women between the ages of 35-54 years, and the average age of diagnosis at around 50 years [5]. By screening with Pap test, cervical cancers or precancerous lesions can be found prior to invasive and metastatic disease. HPV testing can be used alongside the Pap test to identify patients with high-risk HPV that may be more likely to develop cancer. With Pap screening, early detection is possible and cancerous or precancerous lesions can be addressed before progression to the advanced stage. However, some women still present with cervical cancer having received Pap smear screening [10].

We present a 30-year-old female with aggressive cervical cancer with diffuse metastases to several different locations such as brain, lung, kidney, adrenal glands, and pelvic wall. Central nervous system (CNS) metastasis from cervical cancer is rare [11, 12]. She had no abnormal Pap smears in the past and had received the HPV vaccines. CNS metastasis can occur in adenocarcinoma, squamous cell carcinoma, and adenosquamous carcinoma. The risk of brain metastasis is increased when tumors are poorly differentiated, such as in our patient. The route of spread is likely hematogenous, but there are many other factors to consider including tumor emboli and the host immune response [11, 13]. Brain lesions are often supratentorial, likely due to vascularity and spatial characteristics of the area. Patients may present with neurologic symptoms, such as headaches and hemiparesis, depending on the involved area of the brain. In the retrospective study by Hwang et al., the incidence of brain metastasis in the study population was 0.45% with a median age of initial diagnosis of cervical cancer being 50 years. The time from diagnosis of cervical cancer to the identification of brain metastatic was roughly 15.4 months with the median survival time after diagnosis of the brain mets being 5.9 months showing a poor prognosis [14].

While our patient had brain metastases on preliminary staging scans, her initial presentation (including difficulty with ambulation) was due to spinal cord metastasis. Malignant spinal cord compression (MSCC) is most commonly seen in breast, lung, and prostate cancers [15] and occurs in up to 5% of patients with cancer [16]. Our case is unique in that on presentation, the patient had a significant metastatic burden, including spinal cord compression due to thoracic spine metastasis from cervical cancer. It is an emergency that requires prompt intervention, including steroid treatment to reduce inflammation and surgical decompression. Radiation therapy is sometimes utilized as well. The method of treatment is dependent on patient factors, functional status, and is aimed at reducing pain and preserving neurological function. While the incidence of MSCC is unknown, post-mortem studies estimate it occurs in 5-14% of patients with solid tumors [16]. MSCC is usually seen later in life, with the median age of diagnosis being 65 years old. However, this is in regard to any cancer type causing MSCC and not limited to cervical squamous cell carcinoma, as seen in our patient. Initially, the patient had good functional status, and surgical decompression was performed to preserve neurologic function.

Treatment options for metastasis to the brain included surgical resection and radiation therapy. Surgery is usually preferred when there is a solitary lesion. In patients that are poor surgical candidates or have multiple lesions, systemic treatment is preferred [13]. According to Ikeda et al. surgical excision of the brain lesions followed by postoperative radiotherapy had a better survival rate than radiation treatment alone [17]. Palliative therapies have focused on neurological symptom control such as Gamma-knife radiosurgery [18]. Whole brain radiation was attempted in our patient however she was unable to tolerate it.

The patient had an elevated CA-125 which is a protein seen in most ovarian cancer cells; however, the blood levels are also increased in cancers of uterine, fallopian tube, pancreas, breast, lung, and colorectal origin. The patient’s CA27-29 was also elevated and is usually increased in carcinoma of the breast, small cell lung cancer, non-small cell lung cancer along with cancers of liver, colon, pancreatic, gastric, ovarian, and uterine etiology. CA27-29 levels have been used for the clinical management of metastatic breast cancer and monitor therapeutic response [19].

Chemotherapy consisting of platinum-based combination therapy along with an angiogenesis inhibitor such as bevacizumab is the first-line treatment in patients with recurrent, metastatic, or advanced cervical cancer [20]. Our patient was treated with Carboplatin and Paclitaxel based on National Comprehensive Cancer Network (NCCN) guidelines. Usually, the addition of PD-1 inhibitor depends on the PD-L1 status. Pembrolizumab was given inpatient along with chemotherapy as a one-time dose although PD-L1 testing had not been done.

Immunohistochemical examination of the spinal tumors was positive for cytokeratin (CK 5/6) and caudal-type homeobox protein 2 (CDX2) which are important markers for SCC. It was variably positive for p16 protein which is a surrogate marker for HPV infection and is overexpressed in cervical cancers. P16 promotes synthesis of retinoblastoma protein (Rb). To further guide treatment, FoundationOne was planned however the patient passed away.

## Conclusions

The prognosis of cancer that has metastasized to the brain is very poor, but a multi-modal treatment approach, consisting of chemotherapy, surgery, and radiation provides options for prolongation of life. Cervical cancer spreads locally or distantly to organs such as lungs and bones, however, CNS metastasis, especially intracranial metastasis is a rare phenomenon. It usually invades local structures in the pelvis and nearby lymph nodes.

Currently, there is no standard treatment for severe metastatic cervical cancer with CNS involvement. However, surgical resection and radiation treatment have been used for management. Metastatic intracranial cervical cancer has a poor prognosis nevertheless, factors such as age less than 50 years and the number of cerebral lesions are good prognostic factors. In our case, the patient was treated with systemic treatment consisting of Carboplatin, Paclitaxel, and Pembrolizumab with radiation treatment. However, due to her severe disease burden, the patient and her family opted for palliative measures and she passed away shortly thereafter.
